# Plasma proteomic signatures of liver steatosis and fibrosis in people living with HIV: a cross-sectional study

**DOI:** 10.1016/j.ebiom.2024.105407

**Published:** 2024-10-18

**Authors:** Louise E. van Eekeren, Quirijn de Mast, Elise M.G. Meeder, Adriana Navas, Albert L. Groenendijk, Marc J.T. Blaauw, Wilhelm A.J.W. Vos, Nadira Vadaq, Jéssica C. Dos Santos, Joost Rutten, Niels P. Riksen, Jan van Lunzen, Gert Weijers, Mihai G. Netea, André J.A.M. van der Ven, Eric T.T.L. Tjwa, Leo A.B. Joosten

**Affiliations:** a Department of Internal Medicine, Radboud University Medical Centre, Nijmegen, the Netherlands; b Radboud Centre for Infectious Diseases, Radboud University Medical Centre, Nijmegen, the Netherlands; c Department of Medical Microbiology and Infectious Diseases, Erasmus Medical Centre, the Netherlands; d Department of Internal Medicine, OLVG, Amsterdam, the Netherlands; e Medical UltraSound Imaging Centre (MUSIC), Division of Medical Imaging, Radboud University Medical Centre, Nijmegen, the Netherlands; f Department of Metabolism and Immunology, Life and Medical Sciences Institute, University of Bonn, Bonn, Germany; g Department of Gastroenterology and Hepatology, Radboud University Medical Centre, Nijmegen, the Netherlands; h Department of Medical Genetics, Iuliu Hatieganu University of Medicine and Pharmacy, Cluj-Napoca, Romania

**Keywords:** HIV, MASLD, Steatosis, Fibrosis, Proteomics

## Abstract

**Background:**

Insights into the mechanisms driving metabolic dysfunction-associated steatotic liver disease (MASLD) in people living with HIV (PLHIV) remain limited. Plasma proteomics holds promise for biomarker discovery and the elucidation of biological mechanisms.

**Methods:**

We performed cross-sectional analyses on data from 1036 virally suppressed PLHIV using antiretroviral treatment (ART) from the Dutch multi-centre 2000HIV cohort. Participants underwent transient elastography to assess liver steatosis (controlled attenuation parameter (CAP) ≥263 dB/m) and -fibrosis (liver stiffness measurement (LSM) ≥7.0 kPa). Plasma protein concentrations (n = 2367) (Olink® Explore Panel) were compared between PLHIV with vs. without liver steatosis and PLHIV with vs. without fibrosis. Enriched pathways (using GO, KEGG and Reactome libraries) and correlations with clinical characteristics were assessed, and analyses were stratified by BMI category. In addition, concentrations of 242 proteins were compared between individuals (“controls”) with and without liver steatosis (ratio of methylene:methylene and water >5.6% on magnetic resonance spectroscopy) from a separate cohort (300-OB), all having a BMI >26 kg/m^2^.

**Findings:**

Steatosis and fibrosis were associated with 67/2367 (2.2%) and 17/2367 (0.7%) differentially expressed proteins (DEP), respectively, enriched in mostly metabolic pathways. Immunoglobulin superfamily member 9 (IGSF9) was amongst the top DEP associated with both steatosis and fibrosis. Stratifying by BMI revealed 8/2367 DEP associated with steatosis in lean- and 12/2367 DEP in overweight/obese individuals, with two shared DEP (IGSF9 and GHR). Conversely, protein signatures of overweight/obese PLHIV (32/242 DEP) and overweight/obese HIV-uninfected individuals (32/242 DEP) exhibited substantial overlap with 16 shared DEP. Notably, DEP correlated with HIV characteristics in lean individuals but not in overweight/obese PLHIV.

**Interpretation:**

Lean and overweight/obese PLHIV exhibit distinct proteomic signatures associated with liver steatosis, with the former being more strongly correlated with HIV-specific factors and ART. In addition, we identified a protein, IGSF9, strongly related to liver fibrosis and steatosis across BMI categories.

**Funding:**

The 2000HIV study is funded by 10.13039/100010877ViiV Healthcare.

**Research in context**

**Evidence before this study**

Metabolic dysfunction-associated steatotic liver disease (MASLD, formerly known as non-alcoholic fatty liver disease (NAFLD)) has emerged as one of the leading causes of liver disease among people living with HIV (PLHIV), but the exact pathophysiology remains to be elucidated. In a prior analysis of our cohort, we found that factors related to HIV and exposure to antiretroviral treatment (ART) are associated with SLD in lean, but not in overweight or obese PLHIV, suggesting different pathophysiology. High throughput proteomics holds promise to increase understanding of biological mechanisms and has already provided useful insights into the pathophysiology of MASLD in the general population.

We searched the MEDLINE database using any combination of the key words 1) “HIV”, 2) “NAFLD”, “MASLD”, or “steatosis”, and 3) “proteome” or “proteomics” for English manuscripts published up to August 1, 2024, to identify studies that used proteomics to address MASLD in PLHIV. We identified one study that assessed the proteome (n = 183 proteins) of PLHIV with fibrosis, but zero studies that assessed the proteome of PLHIV with steatosis.

**Added value of this study**

Immunoglobulin superfamily member 9 (IGSF9), a cell-adhesion protein that has not yet been described in the context of MASLD, is a top differentially expressed protein associated with fibrosis and steatosis across BMI categories. In addition, lean and overweight/obese PLHIV have distinct proteomic signatures of liver steatosis, with the former being more associated with HIV and ART.

**Implications of all the available evidence**

Our study has revealed a potential biomarker of SLD: IGSF9. Furthermore, our data provide valuable information on the pathophysiology of SLD, pointing towards distinct pathophysiological mechanisms involving HIV-related factors and exposure to ART in lean PLHIV. These findings may aid the diagnosis of SLD in PLHIV.

## Introduction

Metabolic dysfunction-associated steatotic liver disease (MASLD, formerly known as non-alcoholic fatty liver disease (NAFLD)) has emerged as one of the leading causes of liver disease among people living with HIV.^1^ MASLD encompasses a spectrum of conditions, ranging from simple steatosis to metabolic dysfunction-associated steatohepatitis (MASH), fibrosis and cirrhosis, and may eventually lead to hepatocellular carcinoma.^2^ MASLD is closely associated with obesity, but in PLHIV, MASLD represents a significant burden in those with a lean body as well.^3^ In addition to known traditional metabolic risk factors, HIV-associated immune activation and exhaustion and effects of antiretroviral treatment may play a role in the development and progression of MASLD, especially in lean PLHIV.^4^^,^^5^ However, the exact mechanisms that contribute to the development of liver steatosis and fibrosis in PLHIV remain incompletely understood.

High-throughput proteomic technologies have emerged as a valuable tool for the discovery of biomarkers and to increase the understanding of biological mechanisms in various diseases. In the general population, proteomics analyses have revealed protein signatures for different components of SLD.^6^^,^^7^ Such signatures not only aid in unravelling biological mechanisms driving the disease, but also in diagnosis and monitoring disease progression.^7^ For example, the protein ADAMTSL2 was associated with zonal activation of hepatic stellate cells and was revealed as an accurate marker for the detection of fibrosis in MASLD.^7^ To date, proteomics studies on SLD in PLHIV are scarce. No studies have reported plasma proteome association with liver steatosis and only one study investigated its association with liver fibrosis.^8^ In this study, we performed a targeted proteomic analysis of 2367 plasma proteins on liver steatosis and fibrosis in 1036 PLHIV, to gain insights into the underlying pathophysiological mechanisms and to identify possible biomarkers. As mechanisms driving SLD may differ between lean and overweight/obese individuals, analyses were stratified by BMI. In addition, to disentangle the effects of HIV itself, we compared circulating concentrations of a subset of 242 plasma proteins between HIV-uninfected individuals with overweight or obesity and liver steatosis with those individuals without liver steatosis and compared the results to those in PLHIV with overweight or obesity.

## Methods

### Study population

For this proteomics study we used data from two existing cohorts embedded in the Human Functional Genomics Project (HFGP) (http://humanfunctionalgenomics.org).

The longitudinal 2000HIV study comprises 1895 people living with HIV enrolled in four HIV treatment centres in the Netherlands between October 2019 and October 2021.^9^ The total cohort was divided into a discovery (all participants from three of four inclusion sites) and validation cohort (all participants from one of four inclusion sites) to enable immediate validation of findings in an independent cohort. Inclusion criteria were age ≥18 years, use of combination antiretroviral treatment (ART) ≥6 months, and an HIV-1 RNA load <200 copies/mL. Exclusion criteria were detectable viral hepatitis B or C DNA by polymerase chain reaction (PCR), signs of another acute infection, active malignancy, and prior antibiotics use within four weeks. A distinction between lean and overweight/obese PLHIV was made according to BMI: PLHIV with a BMI <25 kg/m^2^ were defined as lean whereas PLHIV with a BMI ≥25 kg/m^2^ were defined as overweight/obese.^10^ Definitions were different in people of Asian descent, were PLHIV with BMI <23 kg/m^2^ were defined as lean and PLHIV with a BMI ≥23 kg/m^2^ as overweight/obese.^11^

As a control cohort, we utilised the cross-sectional 300-OB study comprising 302 participants, aged ≥55 years and with a BMI ≥27 kg/m^2^, and included between 2014 and 2016.^12^ Most of these subjects previously participated in the Nijmegen Biomedical Study, a population-based survey of inhabitants of the Nijmegen municipality, as previously described.^13^ Although participants did not undergo an HIV test, they were recruited from the general population where HIV prevalence rates are below 0.2% and the participants had no signs of immune deficiency. We therefore consider them as HIV-negative controls for this study.

The 2000HIV study was approved by the Independent Review Board Nijmegen (ref. NL68056.091.81) and the 300-OB study was approved by the Ethical Committee of the Radboud University Medical Centre Nijmegen, the Netherlands (ref. 46846.091.13). The study protocol of the 2000HIV study was published at clinicaltrials.gov (ID: NCT03994835). Written informed consent was obtained from all study participants. All experiments were conducted according to the Declaration of Helsinki principles.

### Data extraction

#### 2000HIV

For the analysis of the 2000HIV study we used a cross-sectional design including only data registered or measured at the baseline visit. We extracted demographic data, clinical data including history of HIV and comorbidities, current cART regimens, and comedication from the medical files. CART history, as well as CD4 (nadir, before cART initiation, and latest measurement at enrollment [within 13 months prior to study visit]) and CD8 counts (before cART initiation and at enrollment) and HIV-RNA viral loads (zenith and at enrollment) were obtained from the Stichting HIV Monitoring (SHM, the national Dutch HIV registry. Exposure to dideoxynucleosides, protease inhibitors, and integrase inhibitors as drug classes, and drugs belonging to these classes were expressed as binary variables (no prior exposure vs. at least one year exposure).

Self-reported level of physical activity in general and specifically during work was collected using questionnaires. Total cholesterol, high-density lipoprotein (HDL), low-density lipoprotein (LDL), very low-density lipoprotein (VLDL) cholesterol, and triglycerides were measured in blood obtained during the study visit using a high-throughput nuclear magnetic resonance spectroscopy platform (Nightingale's Biomarker Analysis Platform, Helsinki).^14^

Past hepatitis B infection was defined as presence of hepatitis B surface antibody (anti-HBs) combined with presence of hepatitis B core antibody (anti-HBc).

All information collected from medical files and SHM was collected in electronic case report forms (CRF) in CastorEDC.

Metabolic syndrome was defined using a modified National Cholesterol Education Program definition,^15^ as the presence of ≥ three of the following five traits: 1) abdominal obesity, defined as a BMI ≥30 kg/m^2^ (corresponding to a waist circumference ≥102 cm (40 in) in men and ≥88 cm (35 in) in females^16^; 2) serum triglycerides ≥1.7 mmol/L or lipid-lowering therapy (LLT); 3) serum high-density lipoprotein (HDL) cholesterol <1 mmol/L in males and <1.3 mmol/L in females or LLT; 4) systolic blood pressure ≥130, diastolic blood pressure 85 ≥mmHg or anti-hypertensive medication; and 5) previously diagnosed type 2 diabetes.

#### 300-OB

For the analysis of the 300-OB cohort we extracted demographic data including age, self-reported sex at birth, BMI, and the date of blood collection.

#### Liver measurements in 2000HIV and 300-OB

The liver stiffness measurement (LSM) and controlled attenuation parameter (CAP) were measured in 2000HIV participants using vibration-controlled transient elastography (VCTE) (FibroScan®, Echosens, Paris, France), as previously described.^9^ Cut-off values for steatosis grades were CAP <263 dB/m for S0 (no steatosis), CAP ≥263 dB/m and <280 dB/m for S1 (mild steatosis), and CAP ≥280 dB/m for S2/S3 (moderate to severe steatosis).^17^^,^^18^ Cut-off values for fibrosis grades were LSM <7.0 kPa for F0–F1 (no—mild fibrosis), LSM ≥7.0 and < 8.7 kPa for F2 (moderate fibrosis), as LSM ≥8.7 and < 10.3 kPa for F3 (severe fibrosis), and LSM ≥10.3 kPa for F4 (cirrhosis).^19^ Participants with unsuccessful measurements, liver cirrhosis due to alcohol abuse or viral hepatitis, alcohol abuse in the medical history were excluded from the analyses.^5^

Liver steatosis in 300-OB participants was determined by proton magnetic resonance spectroscopy (MRS) using a 3.0 T Magnetom Skyra or Trio (Siemens, Erlangen, Germany), as previously described.^20^ Intrahepatic triglyceride content was expressed as the fraction of the methylene signal in the combined signal of methylene and water. Liver steatosis was defined as a ratio of methylene: methylene and water >5.6% according to European guidelines.^21^

#### Ultrasound examination of the liver in 2000HIV

We performed standard ultrasonography of the liver using a low-frequency abdominal probe to obtain the “fat layer thickness”, defined as the total thickness of superficial tissue layers (including skin, muscle, subcutaneous and visceral fat) between the transducer and the liver capsule was obtained at the level of the right liver lobe.^9^ Fat layer thickness was dichotomised at a threshold of 25 mm: measurements ≤25 mm were assigned a value of 0, and measurements >25 mm were assigned a value of 1.

#### Carotid artery measurements in 2000HIV

Atherosclerotic plaque(s), measured (as previously described^9^) using ultrasonography, was defined as a focal intima media thickness (IMT) > 1.5 mm or > 50% thickening of the IMT compared with the mean IMT in the common carotid artery, carotid bulb, or internal carotid artery.^22^ Presence of atherosclerotic plaque(s) was used as a marker of subclinical cardiovascular disease and related to the proteomic signature of liver steatosis to assess its relation to comorbidities.

#### Genotyping in the 2000HIV cohort

All individuals were genotyped on the Illumina Infinium Global Screening Array.^9^ After standard quality control procedures, imputation was performed using the TOPMed (version r2 on GRCh38) reference panel on the TOPMed imputation server. After removal of genetic outliers due to excess heterozygosity, relatedness, and ancestry and SNPs with low MAF and imputation quality (MAF >5% and R^2^ > 0.3 or ER^2^ > 0.7 for imputation quality), the genotypes of SNPs of interest (rs738409, rs58542926, rs641738, rs780094 and rs2642438) were extracted for 1022 individuals of European ancestry with liver steatosis or fibrosis data available.

#### Flow cytometry in 2000HIV

Immunophenotyping was performed in blood of participants of the 2000-HIV study using a 21-colour, six-laser CytoFLEX-LX (Beckman Coulter), as previously described.^23^ Daily quality control and standardization were conducted using CytoFLEX Daily QC Fluorospheres (Beckman Coulter, Catalogue #B53230), CytoFLEX Daily IR QC Fluorospheres (Beckman Coulter, Catalogue #C06147), and SPHERO™ Rainbow Calibration Particles 6-Peak (Spherotech Inc, Catalogue # RCP-30-5A-6). Data acquisition was performed with CytExpert software version 2.3 (Beckman Coulter), and data analysis was carried out using a conventional gating strategy in Kaluza software version 2.1.2. For this study, only two populations that represent T cell activation were selected: CD4+HLA-DR+CD38+ and CD8+HLA-DR+CD38+. The percentage and MFI of these populations were used for the correlation analysis with proteomic signatures.

#### Proteomic profiling

Collection, processing, and storage of the samples of the 2000HIV study^9^ and the 300OB study^12^ were performed as previously described. Importantly, processing of samples differed between the two cohorts, hindering direct comparison between participants of the 2000HIV and 300OB cohorts.

Targeted proteomic profiling was performed in plasma samples by Olink® (Olink Bioscience AB, Uppsala, Sweden). Proteins were measured using a proximity extension assay as normalised protein expression levels (NPX) on a log_2_ scale. A total of 3072 plasma proteins (Olink® Explore panel), including inflammatory, cardiometabolic, oncologic, and neurologic proteins, were measured in participants of the 2000HIV study. In the 300-OB cohort, three different panels were measured: Olink® Target 96 Inflammation, Olink® Target 96 Cardiovascular II, and Olink® Target 96 Cardiovascular III, each comprising 92 proteins.

#### Statistical analysis

Binary variables were summarised as percentages and compared between groups using Chi-squared test. Continuous baseline characteristics were summarised as medians and compared using Mann–Whitney U- or Kruskal–Wallis test. Expression levels of proteins were compared between groups using parametric ANOVA.

#### Protein expression profiles

##### Quality control of proteomic data

Quality control (QC) and normalization of proteins measured in participants of the 2000HIV study were performed as previously described.^9^ A total number of 2367 proteins remained after QC for further analysis. Similarly, quality control and normalization were performed for the proteins measured in participants of the 300-OB cohort. QC per sample and normalization was already performed by Olink® in-house services. Proteins with limit of detection (LOD) ≥25 of the samples (n = 21 proteins) were excluded, as well as duplicated proteins (n = 10), resulting in 245 proteins for downstream data analysis. 242 of 245 proteins were also measured in PLHIV. Samples deviating more than four standard deviations from the mean of PC1 or PC2 were considered outliers and excluded (n = 1) (Fig. S1).

##### Confounder selection

The list of potential confounders was based on prior analyses (i.e. age, sex, and fat layer thickness^5^) as well associations between the first five principal components (PCs) of the proteome and potential confounders using a linear regression model in the discovery cohort (i.e. seasonality, and the state of lockdown during the COVID-19 pandemic). Immunological effects of seasonality^24^ and lockdowns (unpublished data) have previously been reported. The results were presented as adjusted R^2^ values, with variables having high adjusted R^2^ values considered as potential confounders (Fig. S2). The list of confounders identified in the discovery cohort was applied to the validation cohort. The same confounders were identified for the models of liver steatosis and fibrosis, across different protein categories. Participants with missing data on these confounders were not included in the differential expression analyses.

##### Differential expression analysis (DEA)

DEA were performed using a linear regression model (Limma R package), correcting for age, sex, fat layer thickness, seasonality, and the state of lockdown during the COVID-19 pandemic. Findings in the discovery cohort were validated using the validation cohort. Proteins with FDR-corrected p-value <0.05 and raw p-value <0.05 were considered statistically significant in the discovery and validation cohort, respectively.

An exception was made for the 300-OB cohort and the matched subset of PLHIV. DEA were adjusted for age, sex, and seasonality, as data on fat layer thickness were not available in the 300-OB participants and all were included before the COVID-19 pandemic. Proteins with FDR-corrected p-value <0.05 were considered statistically significant.

##### Tissue- and cell-specific protein enrichment analysis

Enrichment of tissue- and cell-specific proteins was assessed as previously described.^25^

##### Protein–protein interaction network

Network analysis of DEP was performed using Spearman correlation coefficients. DEP with a rho >0.5 were visualised in a network, where proteins were visualised as nodes, the importance of the protein according to page rank centrality by the colour of the nodes, and strength of the correlation coefficient between proteins by the colour of the edges.

##### Functional pathway enrichment analysis

We performed a functional pathway enrichment analysis of DEP using the online platform Metascape. We assessed enrichment of pathways of the Gene Ontology Biological Processes (n = 2732), Reactome Gene sets (n = 346) and Kyoto Encyclopaedia of Genes and Genomes (KEGG) (n = 258) libraries, with a minimal overlap of three proteins, a p-value cut-off of 0.05, and a minimal enrichment of 1.5.

##### Correlation analysis

DEP were correlated with markers of inflammation (i.e. IL6, CD14, and CD163 from the protein panels), immune activation (i.e. percentage and MFI of CD4+ and CD8+ subsets expressing HLA-DR and CD38), cholesterol metabolism, specific single nucleotide polymorphisms (SNPs), HIV-specific characteristics, prior and current exposure to ART, and comorbidities using pairwise complete Spearman correlation.

Statistical analyses and data visualization were conducted in R version 4.3.0. The graphical abstract and the analysis overviews were created using BioRender.

### Role of funders

The 2000HIV study is funded by ViiV Healthcare. ViiV Healthcare does not play a role in the study design, data collection, data analyses, interpretation, or writing of this manuscript.

## Results

### Characteristics of PLHIV

CAP and/or LSM results were available of 1036 PLHIV; 1022 (98.6%) also had results from plasma proteomics. Of these, 814 PLHIV and 959 PLHIV were included in the steatosis and fibrosis analyses, respectively. Of 814 PLHIV, 286 PLHIV (35.1%) had simple steatosis (≥S1 and F0–F1) whereas 528 PLHIV (64.7%) did not have liver steatosis nor fibrosis (S0 and F0–F1). Of 959 PLHIV, 873 (91.0%) did not have liver fibrosis (F0–F1) whereas 86 PLHIV (9.0%) had liver fibrosis (≥F2), regardless of steatosis degree. The baseline characteristics are shown separately in Table 1 for 2000HIV study participants included in the steatosis and fibrosis analyses. Table S1 depicts the baseline characteristics by discovery and validation cohort.

**Table 1:** Baseline table showing characteristics of 2000HIV participants included in the steatosis (left columns) and fibrosis (right columns) analyses.

Variable	Steatosis				Fibrosis			
	Overall, N = 814	S0, N = 528	S1 or higher, N = 286	p-value	Overall, N = 959	F0–F1, N = 873	F2 or higher, N = 86	p-value
**Female sex**	106 (13%)	75 (14%)	31 (11%)	0.173	128 (13%)	120 (14%)	8 (9.3%)	0.248
**Age**	52 (43, 59)	50 (40, 57)	55 (49, 61)	**<0.0001**	52 (43, 59)	52 (43, 59)	54 (41, 62)	0.462
**Ethnicity**				0.107				0.451
Asian	33 (4.1%)	24 (4.6%)	9 (3.1%)		38 (4.0%)	36 (4.1%)	2 (2.3%)	
Black	83 (10%)	60 (11%)	23 (8.0%)		99 (10%)	94 (11%)	5 (5.8%)	
Hispanic	21 (2.6%)	13 (2.5%)	8 (2.8%)		23 (2.4%)	22 (2.5%)	1 (1.2%)	
Mixed	40 (4.9%)	32 (6.1%)	8 (2.8%)		50 (5.2%)	43 (4.9%)	7 (8.1%)	
Native American	3 (0.4%)	2 (0.4%)	1 (0.3%)		3 (0.3%)	3 (0.3%)	0 (0%)	
White	632 (78%)	395 (75%)	237 (83%)		744 (78%)	673 (77%)	71 (83%)	
**BMI (kg/m2)**	24.9 (22.6, 27.6)	23.9 (21.7, 26.1)	26.8 (24.7, 30.0)	**<0.0001**	25.1 (22.7, 27.8)	24.9 (22.5, 27.5)	27.6 (24.3, 30.8)	**<0.0001**
**BMI classification**				**<0.0001**				**0.000**
Lean	407 (50%)	328 (62%)	79 (28%)		465 (49%)	439 (50%)	26 (30%)	
Overweight or obese	405 (50%)	198 (38%)	207 (72%)		492 (51%)	432 (50%)	60 (70%)	
**Physical activity in general**				**<0.0001**				0.072
Low	132 (19%)	63 (14%)	69 (28%)		161 (20%)	140 (19%)	21 (30%)	
Average	426 (61%)	279 (62%)	147 (60%)		496 (61%)	460 (61%)	36 (51%)	
Above average	136 (20%)	108 (24%)	28 (11%)		161 (20%)	148 (20%)	13 (19%)	
**Physical activity during work**				0.081				0.269
Sedentary occupation	166 (24%)	107 (24%)	59 (24%)		188 (23%)	172 (23%)	16 (23%)	
Sedentary occupation, walk/cycle to work	73 (11%)	42 (9.4%)	31 (13%)		96 (12%)	83 (11%)	13 (19%)	
Often physically active during work	206 (30%)	147 (33%)	59 (24%)		245 (30%)	222 (30%)	23 (33%)	
Physically straining work (lifting)	66 (9.5%)	45 (10%)	21 (8.6%)		75 (9.2%)	70 (9.4%)	5 (7.1%)	
Unemployed or retired	182 (26%)	108 (24%)	74 (30%)		212 (26%)	199 (27%)	13 (19%)	
**Smoker (Current)**	248 (33%)	174 (36%)	74 (28%)	**0.033**	289 (33%)	264 (33%)	25 (32%)	0.919
**Pack years**	5 (0, 23)	5 (0, 22)	5 (0, 23)	0.768	5 (0, 23)	5 (0, 23)	5 (0, 19)	0.832
**CMV serology (Pos. IgG)**	753 (93%)	487 (93%)	266 (93%)	0.693	893 (94%)	811 (93%)	82 (95%)	0.468
**CAP (dB/m)**	243 (212, 281)	221 (198, 242)	295 (278, 323)	**<0.0001**	245 (213, 284)	243 (212, 281)	286 (227, 319)	**<0.0001**
**LSM (kPa)**	4.30 (3.60, 5.18)	4.10 (3.50, 4.80)	4.70 (4.00, 5.50)	**<0.0001**	4.40 (3.70, 5.50)	4.30 (3.60, 5.20)	7.90 (7.30, 9.05)	**<0.0001**
**Hypertension**	199 (24%)	104 (20%)	95 (33%)	**<0.0001**	236 (25%)	213 (24%)	23 (27%)	0.630
**T2DM**	38 (4.7%)	16 (3.0%)	22 (7.7%)	**0.003**	51 (5.3%)	38 (4.4%)	13 (15%)	**0.000**
**Lipid lowering therapy**	159 (20%)	76 (14%)	83 (29%)	**<0.0001**	199 (21%)	173 (20%)	26 (30%)	**0.023**
**Metabolic syndrome**	207 (25%)	82 (16%)	125 (44%)	**<0.0001**	255 (27%)	219 (25%)	36 (42%)	**0.001**
**Prior infection with HAV**	80 (9.8%)	48 (9.1%)	32 (11%)	0.337	93 (9.7%)	86 (9.9%)	7 (8.1%)	0.609
**Prior infection with HBV**	246 (31%)	166 (32%)	80 (28%)	0.239	290 (31%)	267 (31%)	23 (28%)	0.499
**Prior infection with HCV**	62 (7.6%)	42 (8.0%)	20 (7.0%)	0.622	75 (7.8%)	69 (7.9%)	6 (7.0%)	0.760
**HIV duration (Years)**	12 (6, 18)	11 (6, 17)	12 (7, 19)	0.097	11 (6, 17)	12 (6, 18)	10 (5, 15)	0.064
**Transmission route**				0.081				0.504
Blood products	2 (0.3%)	2 (0.4%)	0 (0%)		3 (0.3%)	2 (0.2%)	1 (1.3%)	
Congenital	7 (0.9%)	7 (1.4%)	0 (0%)		8 (0.9%)	8 (1.0%)	0 (0%)	
Heterosexual	178 (23%)	107 (21%)	71 (26%)		212 (23%)	195 (23%)	17 (22%)	
IV drug use	7 (0.9%)	6 (1.2%)	1 (0.4%)		7 (0.8%)	7 (0.8%)	0 (0%)	
MSM	586 (75%)	386 (76%)	200 (74%)		684 (75%)	624 (75%)	60 (77%)	
**Nadir CD4 count (10ˆ6 cells/L)**	0.27 (0.16, 0.41)	0.28 (0.17, 0.41)	0.25 (0.12, 0.41)	0.065	0.27 (0.16, 0.41)	0.27 (0.16, 0.41)	0.26 (0.16, 0.40)	0.887
**CD4:CD8 ratio pre-ART**	0.28 (0.17, 0.48)	0.30 (0.18, 0.50)	0.24 (0.13, 0.40)	**0.001**	0.28 (0.17, 0.47)	0.28 (0.17, 0.48)	0.29 (0.19, 0.37)	0.833
**HIV-1 RNA zenith (copies/ml)**	100,000 (39,600, 274,068)	100,000 (36,800, 225,000)	100,000 (47,625, 345,282)	0.091	100,000 (40,000, 261,000)	100,000 (40,000, 274,062)	115,000 (52,064, 222,847)	0.442
**CD4 at enrollment (10ˆ6 cells/L)**	0.68 (0.51, 0.89)	0.67 (0.50, 0.85)	0.71 (0.55, 0.92)	0.135	0.69 (0.52, 0.89)	0.69 (0.51, 0.90)	0.66 (0.53, 0.83)	0.600
**CD8 at enrollment (10ˆ6 cells/L)**	0.81 (0.60, 1.11)	0.80 (0.59, 1.08)	0.85 (0.62, 1.18)	0.142	0.81 (0.61, 1.12)	0.81 (0.60, 1.12)	0.90 (0.65, 1.15)	0.189
**CD4:CD8 ratio at enrollment**	0.87 (0.59, 1.17)	0.89 (0.64, 1.16)	0.84 (0.55, 1.16)	0.205	0.87 (0.59, 1.17)	0.88 (0.59, 1.17)	0.82 (0.59, 1.15)	0.426
**ART duration (Years)**	9 (6, 15)	9 (5, 14)	10 (6, 16)	**0.018**	9 (5, 15)	9 (6, 15)	8 (5, 12)	0.146
**No ART at enrollment**	13 (1.6%)	8 (1.5%)	5 (1.7%)	0.777	14 (1.5%)	13 (1.5%)	1 (1.2%)	1.000
**Dual therapy at enrollment**	86 (11%)	49 (9.3%)	37 (13%)	0.096	101 (11%)	94 (11%)	7 (8.2%)	0.460
**NRTI in current regimen**	775 (95%)	507 (96%)	268 (94%)	0.140	916 (96%)	833 (95%)	83 (97%)	1.000
**NtRT in current regimen**	512 (63%)	335 (63%)	177 (62%)	0.660	606 (63%)	549 (63%)	57 (66%)	0.534
**NNRTI in current regimen**	295 (36%)	194 (37%)	101 (35%)	0.686	343 (36%)	317 (36%)	26 (30%)	0.262
**PI in current regimen**	72 (8.8%)	41 (7.8%)	31 (11%)	0.140	84 (8.8%)	79 (9.0%)	5 (5.8%)	0.311
**INSTI in current regimen**	470 (58%)	305 (58%)	165 (58%)	0.984	560 (58%)	502 (58%)	58 (67%)	0.074
**PNPLA3 (rs738409)**				0.874				0.663
C C	334 (57%)	208 (57%)	126 (58%)		403 (59%)	363 (59%)	40 (63%)	
G C	216 (37%)	134 (37%)	82 (37%)		244 (36%)	224 (36%)	20 (31%)	
G G	33 (5.7%)	22 (6.0%)	11 (5.0%)		37 (5.4%)	33 (5.3%)	4 (6.3%)	
**TM6SF2 (rs58542926)**				0.964				0.144
C C	506 (87%)	315 (87%)	191 (87%)		586 (86%)	536 (86%)	50 (78%)	
T C	72 (12%)	46 (13%)	26 (12%)		93 (14%)	79 (13%)	14 (22%)	
T T	5 (0.9%)	3 (0.8%)	2 (0.9%)		5 (0.7%)	5 (0.8%)	0 (0%)	
**MBOAT7 (rs641738)**				0.882				0.749
C C	175 (30%)	107 (29%)	68 (31%)		210 (31%)	188 (30%)	22 (34%)	
T C	294 (50%)	184 (51%)	110 (50%)		339 (50%)	310 (50%)	29 (45%)	
T T	114 (20%)	73 (20%)	41 (19%)		135 (20%)	122 (20%)	13 (20%)	
**GCKR (rs780094)**				0.629				0.763
C C	203 (35%)	126 (35%)	77 (35%)		242 (35%)	218 (35%)	24 (38%)	
T C	304 (52%)	194 (53%)	110 (50%)		349 (51%)	319 (51%)	30 (47%)	
T T	76 (13%)	44 (12%)	32 (15%)		93 (14%)	83 (13%)	10 (16%)	
**MTARC1 (rs2642438)**				0.710				0.100
A A	53 (9.1%)	31 (8.5%)	22 (10%)		62 (9.1%)	56 (9.0%)	6 (9.4%)	
A G	226 (39%)	145 (40%)	81 (37%)		271 (40%)	238 (38%)	33 (52%)	
G G	304 (52%)	188 (52%)	116 (53%)		351 (51%)	326 (53%)	25 (39%)	

The baseline characteristics are compared between groups using Chi-squared tests, Kruskal–Wallis rank sum tests, and Fisher's exact tests. Significant differences (defined as p-values < 0.05) are shown in bold. Abbreviations: CAP, controlled attenuation parameter; LSM, liver stiffness measurement; T2DM, type 2 diabetes mellitus; LLT, lipid lowering therapy; MetS, metabolic syndrome; HepA, pas infection with hepatitis A; HepB, past infection with hepatitis B; HepC, past infection with hepatitis C; ART, antiretroviral treatment; NRTI, nucleoside reverse transcriptase inhibitor; NtRTI, nucleotide reverse transcriptase inhibitor; NNRTI, non-nucleoside reverse transcriptase inhibitor; PI, protease inhibitor; INSTI, integrase strand-transfer inhibitor.

BMI, diabetes mellitus type 2, lipid lowering therapy, and metabolic syndrome were associated with both liver steatosis and fibrosis. Additionally, age, physical activity, current smoking, hypertension, CD4: CD8 ratio pre-ART and the duration treatment with ART were associated with steatosis (Table 1).

### Differentially expressed proteins in PLHIV with simple steatosis

**Fig. 1:**
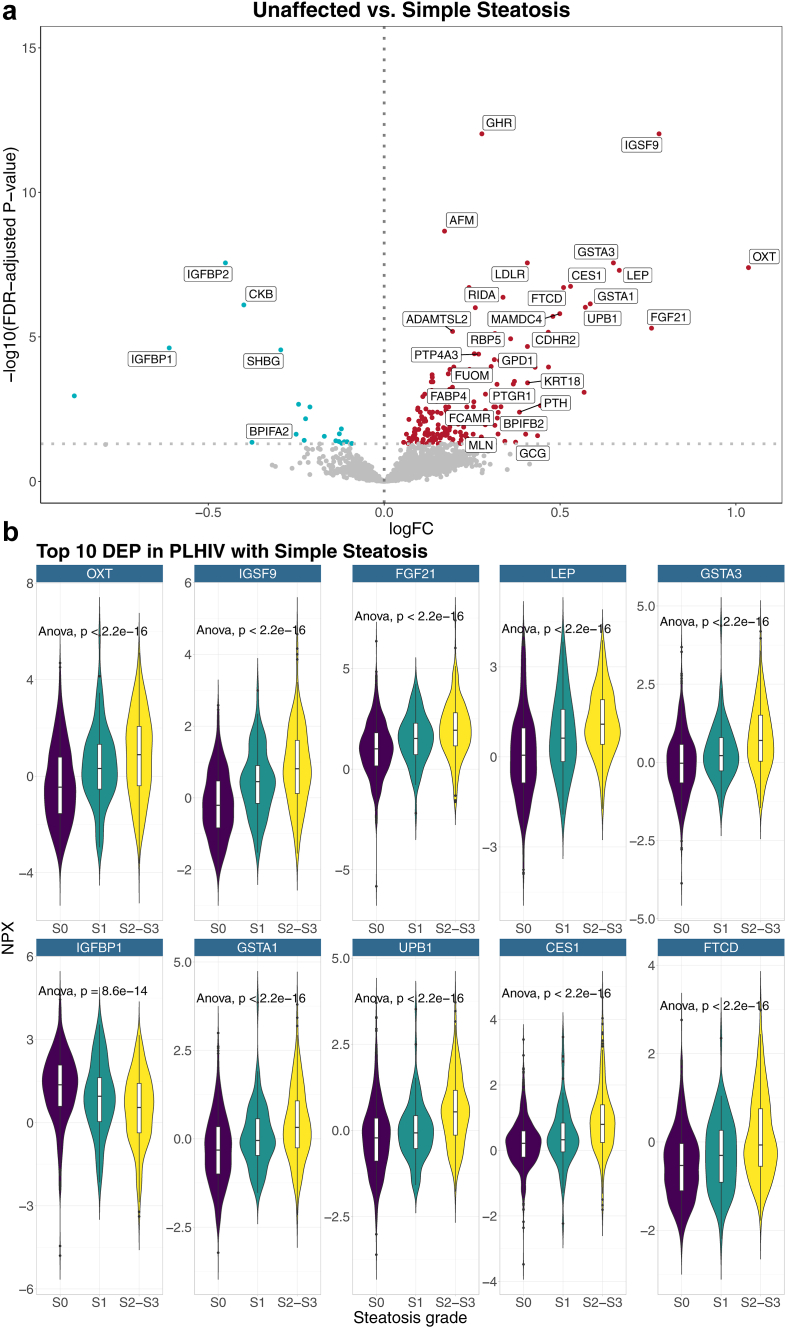
**a** and **b** Differentially expressed proteins in PLHIV with steatosis compared to PLHIV without steatosis. Fig. 1a shows a volcano plot with the results of the DE analysis for the discovery cohort. For readability of the figure, only validated proteins with a logFC < −0.25 or >0.25, or FDR-adjusted p-value <0.00001 in the discovery cohort are annotated. Fig. 1b shows the expression levels (in NPX) of the top 10 proteins by steatosis grade. p-values are derived from comparison by parametric ANOVA tests between each fibrosis grade with correction for multiple testing by FDR.

First, we compared protein concentrations between PLHIV with and without steatosis. A total number of 67/2367 (2.8%) (Fig. 1a, Table S2, Graphical Abstract), including 58 up- and 9 downregulated proteins, were DE. The ten proteins with the highest log fold change were oxytocin (OXT), immunoglobulin superfamily member 9A (IGSF9), fibroblast growth factor 21 (FGF21), leptin (LEP), glutathione S-transferase A3 (GSTA3), insulin-like growth factor-binding protein 1 (IGFBP1), glutathione S-transferase A1 (GSTA1), beta-ureidopropionase (UPB1), liver carboxylesterase 1 (CES1), and formimidoyltransferase-cyclodeaminase (FTCD) (Fig. 1b). IGSF9 and growth hormone receptor (GHR) were the most statistically significant proteins. Most top DEP were involved in glucose-, lipid- and/or amino acid metabolism. FGF21, OXT, LEP, and IGFBP1 have diverse functions including regulation of glucose and lipid metabolism. CES1 is an enzyme involved in lipid and lipoprotein metabolism and FTCD is involved in metabolism of the amino acid histidine and binds and promotes binding of vimentin filaments. In addition, GSTA1 and GSTA3 protect against oxidative stress, UPB1 catalyzes a reaction in the pyrimidine degradation pathway, and IGSF9 is involved in cell-adhesion.

Tissue enrichment analysis indicate that up- and downregulated proteins were mostly enriched in the liver (Fig. S3 and Tables S3–S6). Enriched pathways in PLHIV with steatosis include metabolic pathways such as ‘response to lipoprotein particle’ and ‘regulation of glucose import’, as well as other pathways such as ‘regulation of growth’, ‘regulation of Complement cascade’, ‘cellular response to peptide hormone stimulus’, and ‘drug metabolism’ (Fig. S4 and Table S7).

Next, we assessed interactions between our significant proteins (Fig. S5). In a protein–protein interaction network, IGSF9 was found to have a central position, being correlated to FGF21, CES1, MAMDC4, and afamin (AFM) in PLHIV with steatosis, and to AFM and GHR in those without steatosis.

### Differentially expressed proteins in PLHIV with fibrosis

**Fig. 2:**
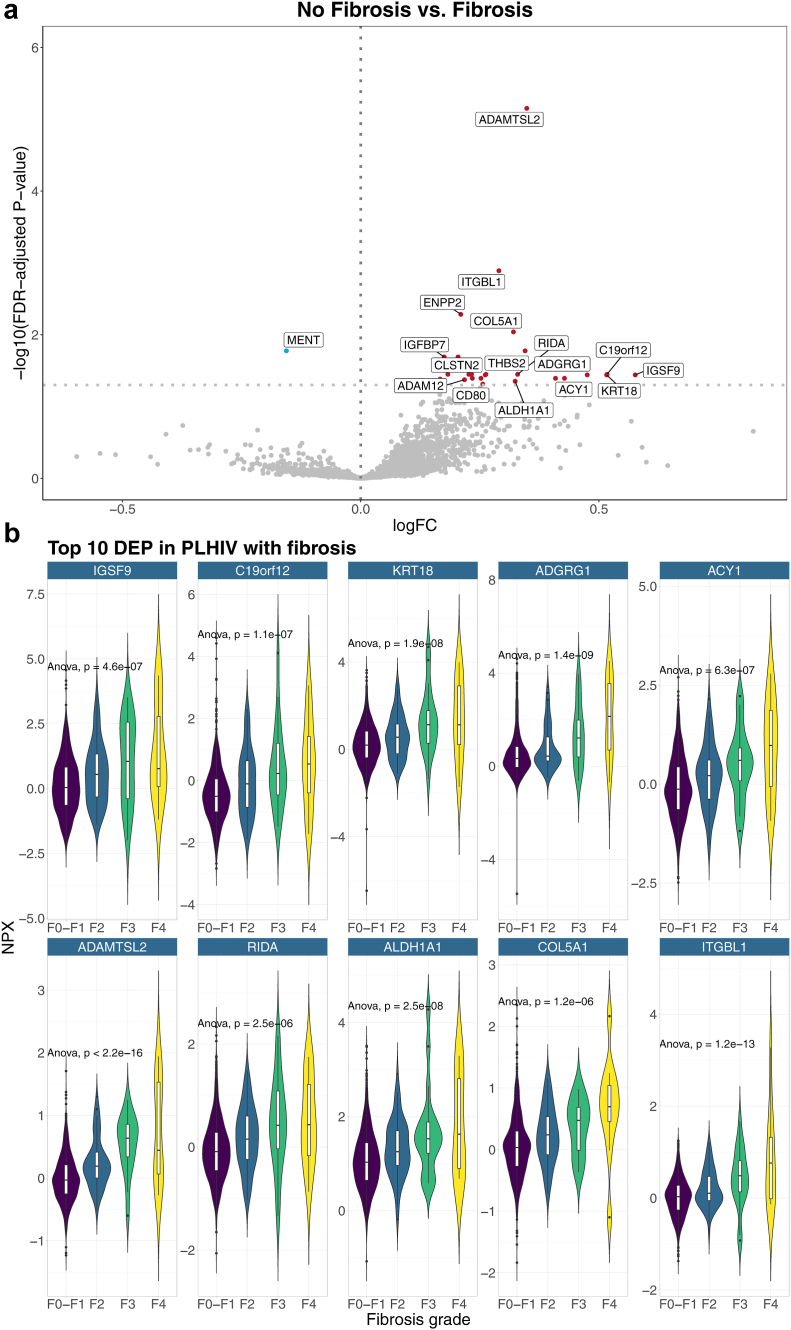
**a** and **b** Differentially expressed proteins in PLHIV with fibrosis compared to PLHIV without fibrosis. a) Volcano plot showing the results of the DE analysis in the discovery cohort with validated proteins annotated. All validated proteins (i.e. FDR-adjusted p-value <0.05 in the discovery- and raw p-value <0.05 in the validation cohort) are annotated. b) Expression levels (in NPX) of the top 10 proteins (as defined by log fold change in discovery cohort) by steatosis grade. p-values are derived from comparison by parametric ANOVA tests between each fibrosis grade with correction for multiple testing by FDR.

Next, we compared protein levels between PLHIV with and without liver fibrosis. A total number of 17/2367 (0.7%) proteins including 16 up- and 1 downregulated protein were DE in PLHIV with fibrosis (Fig. 2a and b, Table S8, Graphical Abstract). The ten proteins with the highest log fold change were IGSF9, C19orf12, cytokeratin-18 (KRT18), adhesion G-protein coupled receptor G1 (ADGRG1), aminoacylase-1 (ACY1), A disintegrin and metalloproteinase with thrombospondin motifs like 2 (ADAMTSL2), 2-iminobutanoate/2-iminopropanoate deaminase (RIDA), retinal dehydrogenase 1 (ALDH1A1), collagen alpha-1 (V) chain (COL5A1), and integrin beta-like protein (ITGBL1). Most of these top DEP play a role in extracellular matrix organization and cell adhesion: ADAMTSL2, ITGBL1, COL5A1 and possibly C19orf12 are extracellular matrix proteins involved in tissue integrity and organization, KRT18 plays a role in filament organization, FTCD binds and promotes binding of vimentin filaments, and IGSF9 and ADGRG1 are involved in cell-adhesion. In addition, ACY1 and RIDA are involved in amino acid metabolism and ALDH1A1 is involved in lipid peroxidation. ‘Methylated in normal thymocytes’ (MENT) was the only downregulated protein. MENT interacts with DNA methylation through DNA methyl transferase 3B (DNMT3B). The expression levels of each protein by fibrosis grade are shown in Fig. 2b: the upregulated DEP incrementally increased while downregulated DEP MENT incrementally decreased by fibrosis stage.

Upregulated DEP were enriched in Leydig cells and fibroblasts, but not in any specific tissue (Fig. S6 and Tables S9 and 10). The number of downregulated DEP (n = 1) in PLHIV with fibrosis was not sufficient for tissue- or cell enrichment analysis. Enriched pathways in PLHIV with fibrosis include metabolic pathways (i.e. ‘Diseases of metabolism’ and ‘amino acid metabolic process’) and ‘regulation of synapse organization’ (Fig. S4).

Of note, IGSF9, ADAMTSL2, KRT18, and RIDA were associated with both steatosis and fibrosis.

**Fig. 3:**
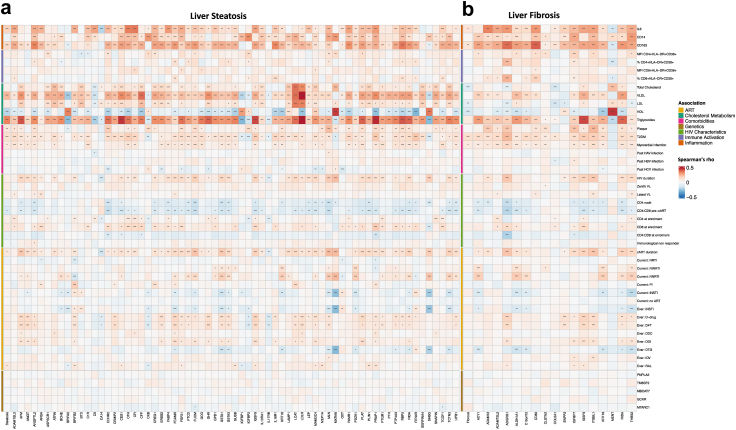
**a** and **b** Correlation between the proteins associated with steatosis (a) and fibrosis (b), with SNPs of interest, markers of inflammation, -immune activation, -cholesterol metabolism, HIV-characteristics, ART exposure, and cardiometabolic diseases. The colours show the direction and strength of the Spearman correlations, and asterixis refer to the FDR-adjusted p-value: ∗ = p-value 0.01–0.05; ∗∗ = p-value 0.001–0.01; ∗∗∗ = p-value <0.001.

Interestingly, several proteins associated with steatosis and fibrosis were significantly correlated with markers of cholesterol metabolism (VLDL, HDL, triglycerides), inflammation (CD14, CD163, IL6), as well as cardiometabolic diseases, HIV-specific parameters, and ART (Fig. 3a and b), but not with SNPs. For example, ADGRG1 and IGSF9, both upregulated in PLHIV with fibrosis, were positively correlated with T2DM, presence of carotid plaques, prior myocardial infarction, duration of HIV and ART, and prior exposure to D-drugs and INSTI (Fig. 3b).

### Proteomic signatures of simple steatosis differ between lean and overweight/obese PLHIV

To investigate whether lean and overweight and obese PLHIV have the same protein disturbances associated with simple steatosis, we separately compared protein concentrations between unaffected PLHIV and PLHIV with simple steatosis within each BMI group. Additionally, we examined correlations between DEP and HIV specific parameters and ART within each BMI group.

**Fig. 4:**
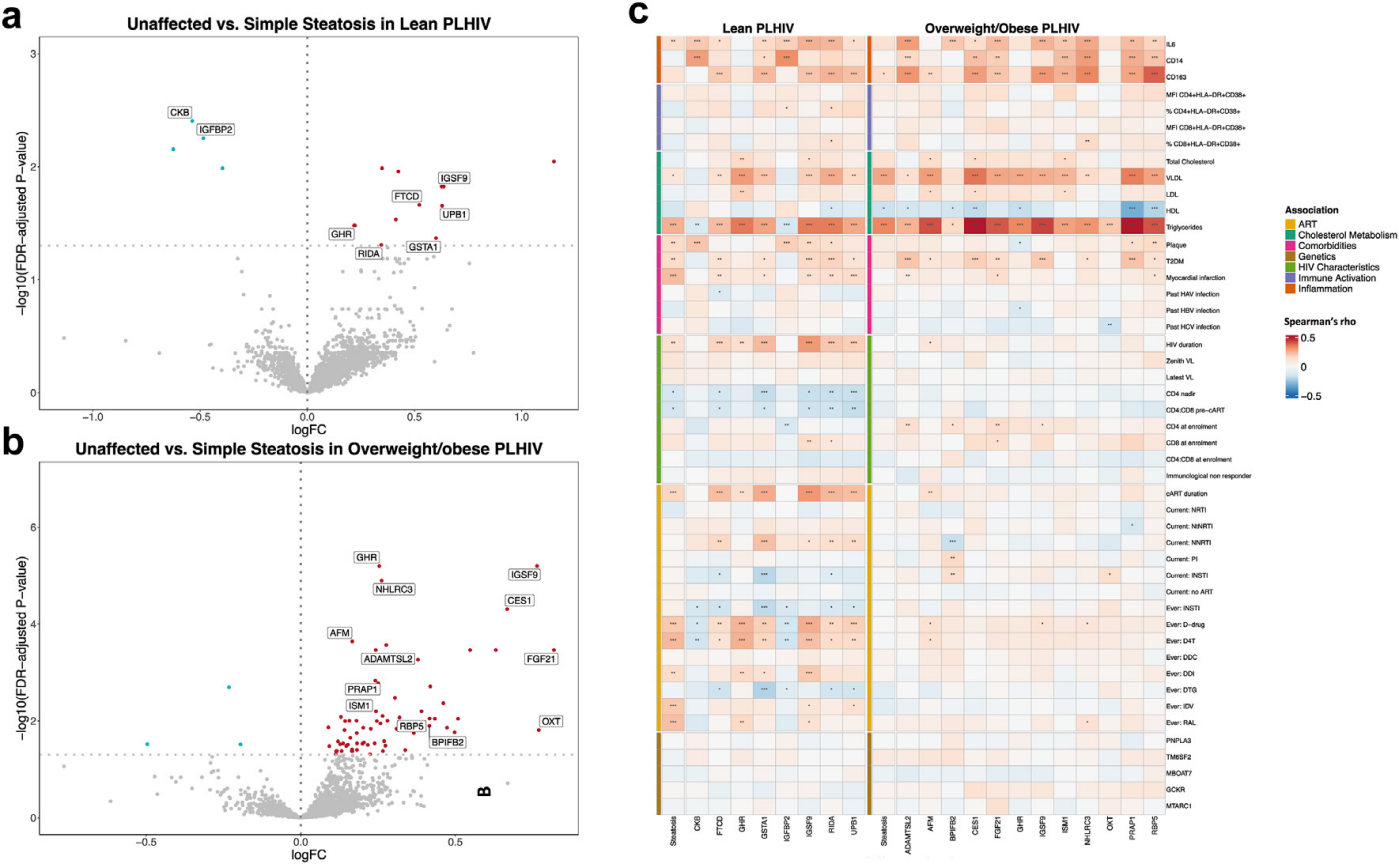
**a–c** Differentially expressed proteins in lean and overweight/obese PLHIV with steatosis compared to PLHIV without steatosis. a) Volcano plot showing the results of the DE analysis lean PLHIV, with only validated DEP (i.e. FDR-adjusted p-value <0.05 in the discovery- and raw p-value <0.05 in the validation cohort) annotated. b) Volcano plot showing the results of the DE analysis in overweight and obese PLHIV, with only validated DEP (i.e. FDR-adjusted p-value <0.05 in the discovery- and raw p-value <0.05 in the validation cohort) annotated. c and d) Correlation between the DEPs associated with steatosis identified in lean PLHIV (left) and overweight/obese PLHIV (right) and SNPs of interest, markers of inflammation, -immune activation, -cholesterol metabolism, HIV-characteristics, ART exposure, and cardiometabolic diseases. The colours show the direction and strength of the Spearman correlations, and asterixis refer to the significance level: ∗ = p-value 0.01–0.05; ∗∗ = p-value 0.001–0.01; ∗∗∗ = p-value <0.001. Abbreviations: D-drug = dideoxynucleoside analogue drugs (includes D4T, DDI, and DDC), D4T, stavudine; cART, combination antiretroviral treatment; NtRTI, nucleotide reverse transcriptase inhibitor; NNRTI, non-nucleoside reverse transcriptase inhibitor; VL, HIV-1 RNA viral load; immunological non resp, immunological non-responder; IDV, indinavir; RAL, raltegravir, T2DM, type 2 diabetes mellitus; DDI, didanosine; DTG, dolutegravir; INSTI, integrase strand transfer inhibitor; DDC, zalcitabine; PI, protease inhibitor; NRTI, nucleoside reverse transcriptase inhibitor; HBV, hepatitis B virus; HAV, hepatitis A virus; HCV, hepatitis C virus.

Fig. 4 shows the results of the DEA in lean and overweight/obese PLHIV respectively. A total of 8/2367 (0.3%) proteins were DE in lean PLHIV (Fig. 4a, Table S11, Graphical Abstract): IGSF9, GHR, UPB1, RIDA, GSTA1, and FTCD were up- and IGFBP2 and CKB were downregulated. A few more (n = 12) proteins were DE in overweight and obese PLHIV (Fig. 4b, Table S12, Graphical Abstract): IGSF9, GHR, RBP5 (retinol-binding protein 5), proline-rich acidic protein 1 (PRAP1), OXT, NHL repeat-containing protein 3 (NHLRC3), isthmin-1 (ISM1), FGF21, CES1, BPI fold-containing family B member 2 (BPIFB2), AFM, and ADAMTSL2 (all upregulated). Overlap between lean and overweight/obese was limited with only two shared DEP (IGSF9 and GHR). Moreover, log fold changes of the 2367 proteins were weakly correlated (Spearman's rho = 0.26, p-value = < 0.0001) between lean and overweight/obese PLHIV in the discovery cohort.

Amino acid metabolic pathways (involving RIDA, UPB1, and FTCD) were enriched in lean PLHIV (Table S13), whereas no enriched pathways were identified in overweight/obese PLHIV. In both subgroups, the number of DEP was not sufficient for tissue- or cell enrichment analysis.

We next assessed correlations between DEP with markers of inflammation, immune activation, cholesterol metabolism, SNPs of interest, HIV-specific characteristics, and comorbidities separately in lean and overweight/obese PLHIV (Fig. 4c). While, DEP in overweight/obese PLHIV were mostly correlated with cholesterol metabolism and inflammation markers, DEP in lean PLHIV were additionally correlated to HIV-related characteristics, exposure to cART, and cardiometabolic diseases. This also accounted for the shared DEP (IGSF9 and GHR). For example, IGSF9 correlated in lean PLHIV with HIV- and cART duration, CD4 nadir, CD4:CD8 ratio pre-cART, CD8 at enrollment, current treatment with NNRTI, exposure to raltegravir, prior exposure to D-drugs, myocardial infarction, T2DM, and presence of carotid plaque, whereas associations with HIV-characteristics and ART were very limited in overweight/obese PLHIV.

### Steatosis signatures are largely similar between overweight and obese PLHIV and overweight and obese controls

Considering the significant correlations between DEP and HIV-specific factors and ART, especially in lean PLHIV, we aimed to assess whether proteomic signatures of steatosis in PLHIV differ from participants in the overweight/obese control cohort (all with a BMI >26 kg/m^2^). Likewise, a subset was created of PLHIV with a BMI >26 kg/m^2^. The subset of controls (n = 254) consisted mostly of males (n = 145, 57%), with a median age of 66 (IQR: 62–70), and a median BMI of 30.0 (IQR: 28.3–31.9). The subset of PLHIV consisted of 317 (82%) males, with a median age of 53 (IQR: 45–60), and a median BMI of 28.7 (IQR: 27.2–30.9).

**Fig. 5:**
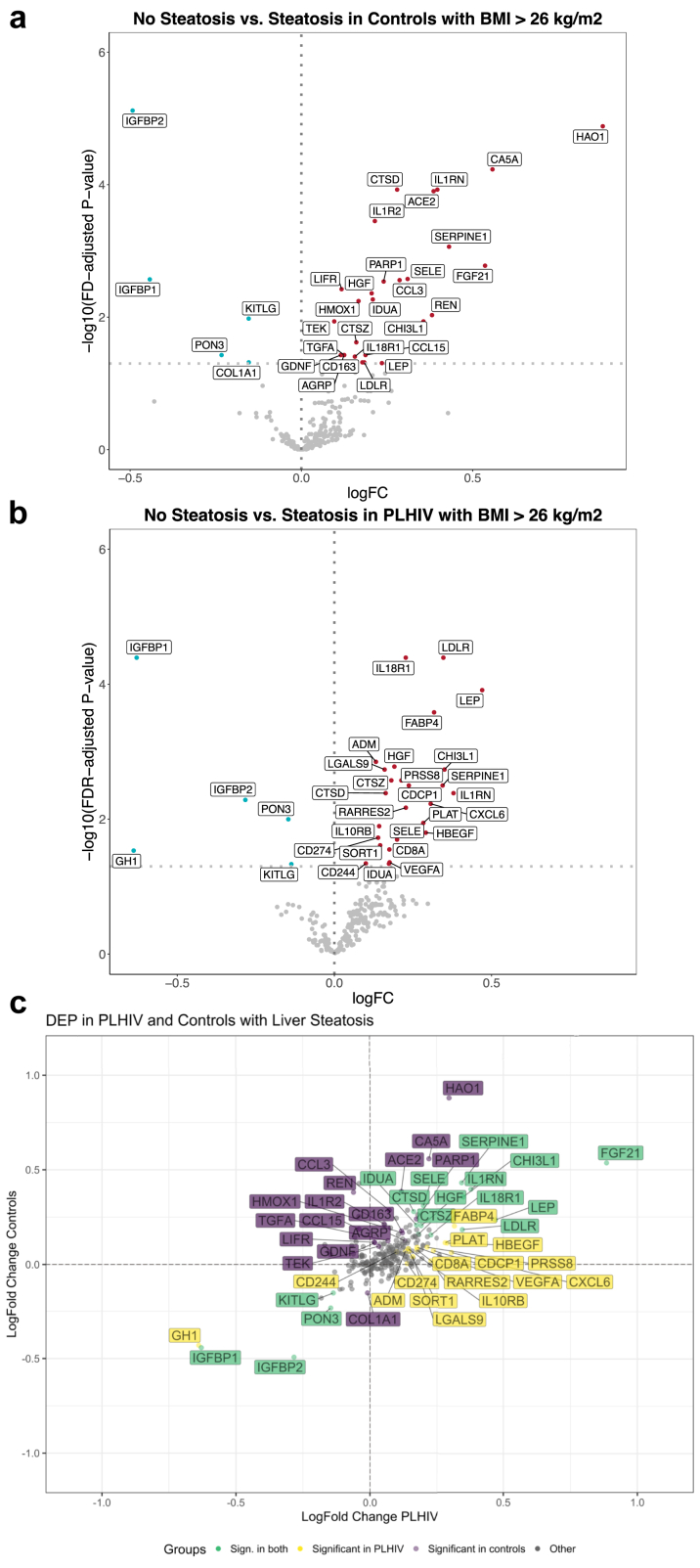
**a–c** DEPs associated with steatosis identified in overweight/obese PLHIV and controls. a) Volcano plot showing the results for overweight/obese controls and b) Volcano plot showing the results for controls. All proteins with an FDR-adjusted p-value <0.05 are annotated. Figure c shows the differences and similarities of the proteomic signature of steatosis between PLHIV and controls.

We compared the expression of 242 inflammatory and cardiovascular proteins between A) controls with (n = 155) vs. without (n = 99) liver steatosis, and B) PLHIV with (n = 233) vs. without (n = 153) liver steatosis, revealing 32 DEP in both PLHIV and controls (Fig. 5a and b (Tables S14 and 15, Graphical Abstract). Sixteen DEP were shared between PLHIV and controls, including 12 up- (FGF21, LEP, IL1RN, CHI3L1, LDLR, SERPINE1, IL18R1, SELE, HGF, CTSZ, IDUA, and CTSD) and 4 downregulated proteins (IGFBP1, IGFBP2, KITLG, and PON3). Among the 16 proteins exclusively associated with steatosis in PLHIV, namely FABP4, CXCL6, HBEGF, PLAT, CPCP1, RARRES2, PRSS8, VEGFA, CD8A, LGALS9, SORT1, IL10RB, CD274, ADM, CD244, and GH1, a consistent trend was observed in controls (Fig. 5c). Likewise, proteins exclusively linked with steatosis in controls exhibited uniform log fold change directions in PLHIV. Accordingly, log fold changes of all 242 proteins were significantly correlated (Spearman's rho = 0.55, p-value <0.0001) between in PLHIV and controls.

## Discussion

Metabolic dysfunction-associated steatotic liver disease (MASLD) is highly prevalent in PLHIV, but proteomics studies are lacking. In the present study we comprehensively assessed the plasma proteomic signatures of liver steatosis and fibrosis in virally suppressed PLHIV using ART, compared proteomic signatures of liver steatosis between lean and overweight/obese PLHIV, and between overweight/obese PLHIV and overweight/obese controls. We identified the protein IGSF9 as a key protein associated with steatosis and fibrosis in PLHIV. In addition, we found different proteomic signatures of steatosis in lean compared to overweight/obese PLHIV. Importantly, DEP in lean PLHIV were correlated with comorbidities, HIV-specific factors, and previous and current ART regimen, while only minor correlations were found with clinical factors for DEP in overweight/obese PLHIV. Hence, our findings provide further evidence for different pathogenic mechanisms driving liver steatosis in lean compared to overweight/obese PLHIV, with more involvement of HIV-specific characteristics and ART in lean MASLD.

IGSF9 was amongst the top DEP for fibrosis and steatosis across BMI categories. IGSF9 concentrations gradually increased by steatosis grade and correlated with prior myocardial infarction, presence of carotid plaques, T2DM, HIV-specific characteristics and prior and current ART. Moreover, IGSF9 has a central position in the protein–protein network of DEP in PLHIV with steatosis. Nonetheless, IGSF9 has not yet been studied in the context of MASLD. IGSF9 is a member of the immunoglobulin superfamily, a class of proteins involved in cell adhesion, binding, and recognition processes. Consequently, IGSF9 is thought to be involved in cell–cell and cell-extracellular matrix (ECM) adhesion.^26^ Previous research on IGSF9 mostly focused on IGSF9 expression in different malignancies. In addition, it has been shown that IGSF9 expression is increased in adipose tissue of HIV-uninfected individuals with type 2 diabetes mellitus,^27^ and IGSF9 gene expression was found to be upregulated in PBMCs of HIV/HCV coinfected individuals after interferon-based treatment compared to gene expression levels before treatment initiation.^28^

It is tempting to speculate on the mechanism linking IGSF9 to MASLD. We found that IGSF9 is significantly correlated with markers of cholesterol metabolism including VLDL and triglyceride levels, as well as markers of inflammation including IL6, CD14, and CD163. Because of the cross-sectional nature of our study, we cannot draw conclusions on causality but can hypothesise based on existing literature. First, like other adhesion molecules, IGSF9 may facilitate the adhesion of immune cells, promoting inflammation, by which hepatic stellate cells become activated and MASLD progresses.^29^ Second, the relationship between IGSF9 and MASLD may rely on the effects of molecular pathways that are modulated by Focal Adhesion Kinase (FAK). FAK, a protein tyrosine kinase, has been shown to play an important role in the progression of liver fibrosis.^30^ FAK becomes activated when ECM proteins bind to integrins. The activation of FAK may be further enhanced upon binding of growth factors to growth factor receptors. ^31^ Notably, our protein–protein network revealed a strong correlation between IGSF9 and growth hormone receptor (GHR). Upon activation, FAK regulates the development of liver fibrosis by several mechanisms, including the activation of hepatic stellate cells (HSC), differentiation of myofibroblasts, cell migration and survival and expression of ECM proteins.^30^ On the other hand, FAK signalling may also attenuate liver fibrosis. It has been shown that mice with FAK-deficient hepatocytes exhibited increased fibrogenesis, which was linked to the hedgehog/smoothened pathway amongst other pro-fibrotic pathways.^32^ In patients with breast cancer, it was shown that IGSF9 interacts with FAK, which led to inhibition of downstream effects of the FAK/AKT signalling pathway including epithelial mesenchymal transition.^31^ Both scenarios suggest that the associations between IGSF9 and cholesterol and triglycerides are indirect and reflect the effects of liver steatosis on lipid metabolism, whereas the link between IGSF9 and inflammation may be more direct and causative of liver steatosis. The link between IGSF9 and liver steatosis should be further investigated.

The second main finding of our study concerns the differences between lean and overweight/obese PLHIV. This is particularly interesting since the pathophysiology of lean MASLD is poorly understood but represents a significant burden in PLHIV.^3^

First, we found that of eight DEP in lean PLHIV with steatosis, and twelve DEP in overweight/obese PLHIV with steatosis, two DEP were shared. This limited overlap indicates distinct pathophysiology between lean and overweight/obese PLHIV. In lean PLHIV with liver steatosis, DEP are involved in insulin sensitivity (i.e. IGFBP-2), lipid metabolism (i.e GHR and CKB), and inflammation and detoxification processes (i.e. GSTA1 and FTCD). In overweight/obese PLHIV with liver steatosis, DEP are involved in lipid metabolism (i.e. RBP5 and FGF21), inflammation and detoxification processes (i.e. PRAP1, CES1, BPIFB2, and AFM), fibrosis (i.e. ADAMTSL2), and hormonal regulation (i.e. OXT). The unique proteomic signatures of lean and overweight/obese PLHIV with liver steatosis suggest differences in multiple mechanisms that may contribute to liver steatosis.

Second, associations of DEP with comorbidities, HIV characteristics, and exposure to ART differed between lean and overweight/obese PLHIV. While DEP in overweight/obese PLHIV with liver steatosis were linked to inflammation and cholesterol metabolism with no apparent association with HIV or ART, DEP in lean PLHIV with liver steatosis were associated not only with inflammation and cholesterol metabolism, but also with comorbidities, HIV-characteristics, and exposure to ART. This accounted as well for the shared DEP.

Third, in a comparison with overweight/obese controls recruited from the general Dutch population, proteomic signatures of steatosis were largely similar between overweight/obese PLHIV and controls.

Altogether, our observations of similar proteomic signatures of steatosis among overweight individuals with or without HIV, with minor correlations with HIV-related factors and ART, in contrast with a unique proteomic signature in lean PLHIV with steatosis with marked correlations with HIV-related factors (i.e. immunological non-responders, CD4:CD8 ratio pre-ART) and exposure to ART (i.e. dideoxynucleosides and INSTI) suggest that mechanisms related to HIV and ART contribute to liver steatosis in lean PLHIV. We cannot explain all associations using the current literature, but we may speculate about some associations: immunological non-responders exhibit persistent inflammation and immune activation, and (prior) treatment with D-drugs such as stavudine may impair mitochondrial function causing decreased beta-oxidation of fatty acids, as well as insulin resistance.^33^^,^^34^ Inflammation, insulin resistance, and alterations in fatty acid metabolism are well-known contributors to MASLD.^35^

This study has a few limitations. First, the cross-sectional nature hinders us to draw conclusions on causality. Second, liver biopsies were not available. Hence, we do not have information on the frequency of steatohepatitis in our population and its proteomic signature. Third, we measured the circulating proteomic signatures and were not able to relate this to protein abundance in the liver itself. Fourth, this study included a low proportion of females. We corrected for the effects of sex to avoid any confounding effect. Fifth, the validation cohort included a higher proportion of participants with liver fibrosis than the discovery cohort. This may have increased statistical power on one hand, but also reduced generalizability on the other.

Finally, few limitations specifically concern our comparison with the control population. First, we were only able to analyse a limited number of proteins. Second, HIV testing was not done in the overweight/obese controls. However, they were recruited from the general population where HIV prevalence rates are below 0.2% and subjects had no sign of immune deficiency. Third, steatosis was assessed in controls using a different modality, namely magnetic resonance spectroscopy. Direct comparison of the two cohorts was not a possibility. Despite these limitations of using the 300-OB cohort as a control cohort, there are some benefits as well. The relation between liver steatosis, as diagnosed by validated methods, and levels of 242 proteins measured by the same methods across both cohorts provides an initial indication of overlapping proteomic signatures between overweight or obese PLHIV and controls.

The main strength of our study is the large proteomics panel consisting of 2367 proteins that we employed in more than 1000 well-characterised PLHIV with transient elastography measurements. Such a study has not been performed previously and has yielded relevant insights into the pathophysiology of MASLD in lean and overweight or obese PLHIV, and possibly interesting biomarkers.

In conclusion, we identified specific proteomic signatures for PLHIV with simple steatosis and for those with fibrosis, including the key protein IGSF9. Proteomic signatures of lean and overweight/obese PLHIV differed with more involvement of HIV-specific factors and ART in lean PLHIV. These insights may be relevant to screening and treatment of MASLD in PLHIV.

## Contributors

LvE, QdM, ET, and LJ contributed to the study design. LvE, EM, AN, AG, MB, and WV performed the investigation. LvE drafted the manuscript. QdM, EM, AN, AG, MB, WV, NV, JDS, JR, NR, JvL, GW, MN, AvdV, ET, and LJ revised and approved the manuscript. LvE, AG, MB, WV, and NV accessed and verified the data. All authors read and approved the final version of the manuscript and had final responsibility for the decision to submit the manuscript for publication.

## Data sharing statement

Data used in this study will be made available with restricted access either via Radboudumc Research or a data repository that is most appropriate for the data when all foreseen manuscripts from the 2000HIV study have been published as it is a collaborative effort in which we re-use the same datasets. Further information and request for data resources should be directed to 2000HIV study principal investigator André J.A.M. van der Ven and project data manager Vasiliki Matzaraki.

## Declaration of interests

The authors have nothing to disclose.

## Appendix A Supplementary data

Supplementary Figures

Graphical Abstract
