# Supplementary material for: Plasma proteomic signatures of liver steatosis and fibrosis in people living with HIV: a cross-sectional study
Source: eBioMedicine. 2024 Oct 18;109:105407. doi: 10.1016/j.ebiom.2024.105407 (PMC11513669; doi:10.1016/j.ebiom.2024.105407)
Supplement: Supplementary Figures [file mmc2.docx]

**Supplementary Figures**

**Fig. S1** PCA plot of proteomic data in 300-OB for outlier detection. Samples deviating more than four standard deviations from the mean of principal component 1 or 2 were considered outliers.

**Fig. S2:** Principal component analysis on potential confounders of the proteome. In addition to the variables age, sex, and fat layer thickness that were previously found to be associated with liver steatosis and fibrosis, genetics (genetic PC1), enrollment during COVID-19 pandemic and seasonality appeared to influence the proteome and were therefore corrected for in the analyses.


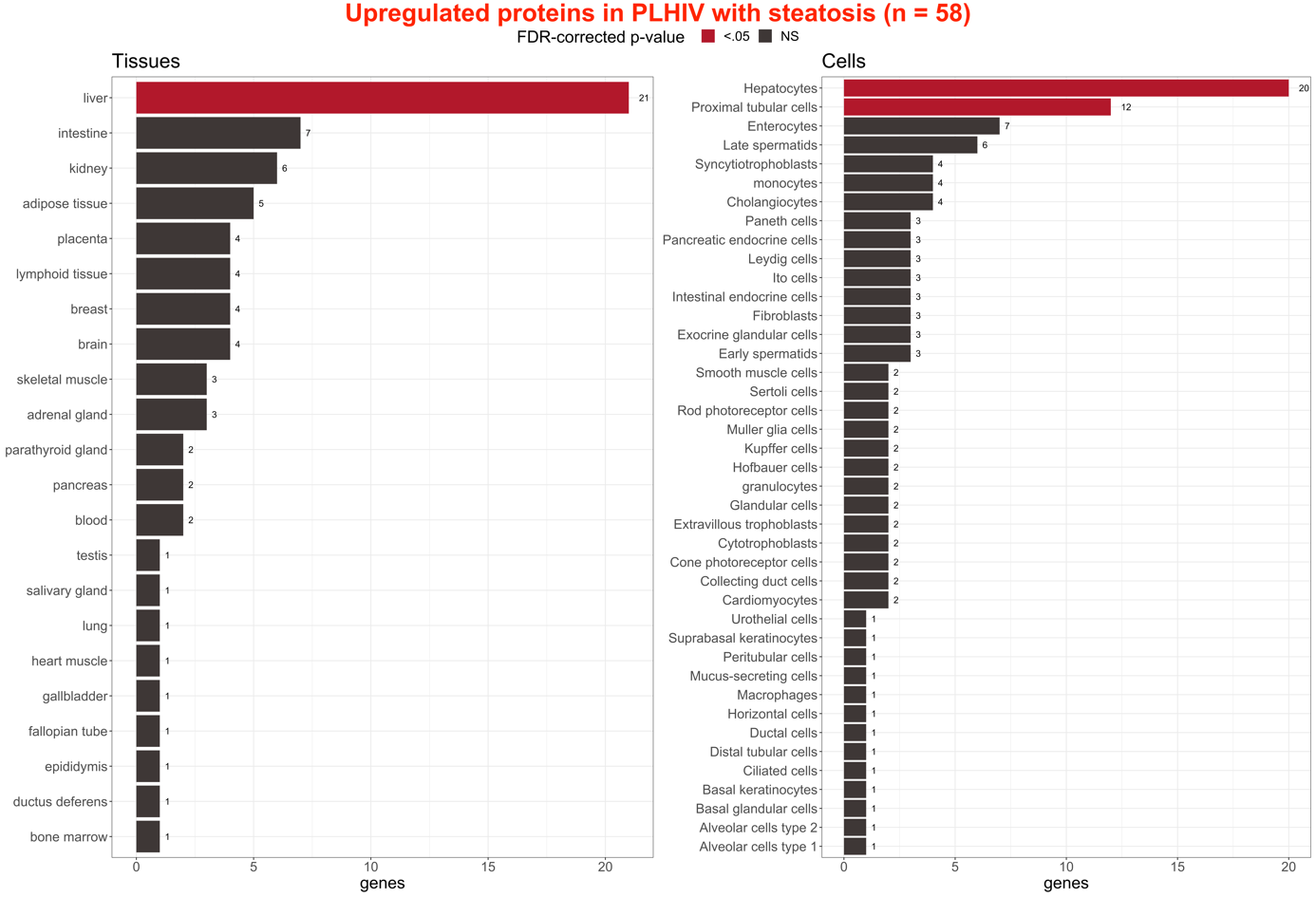


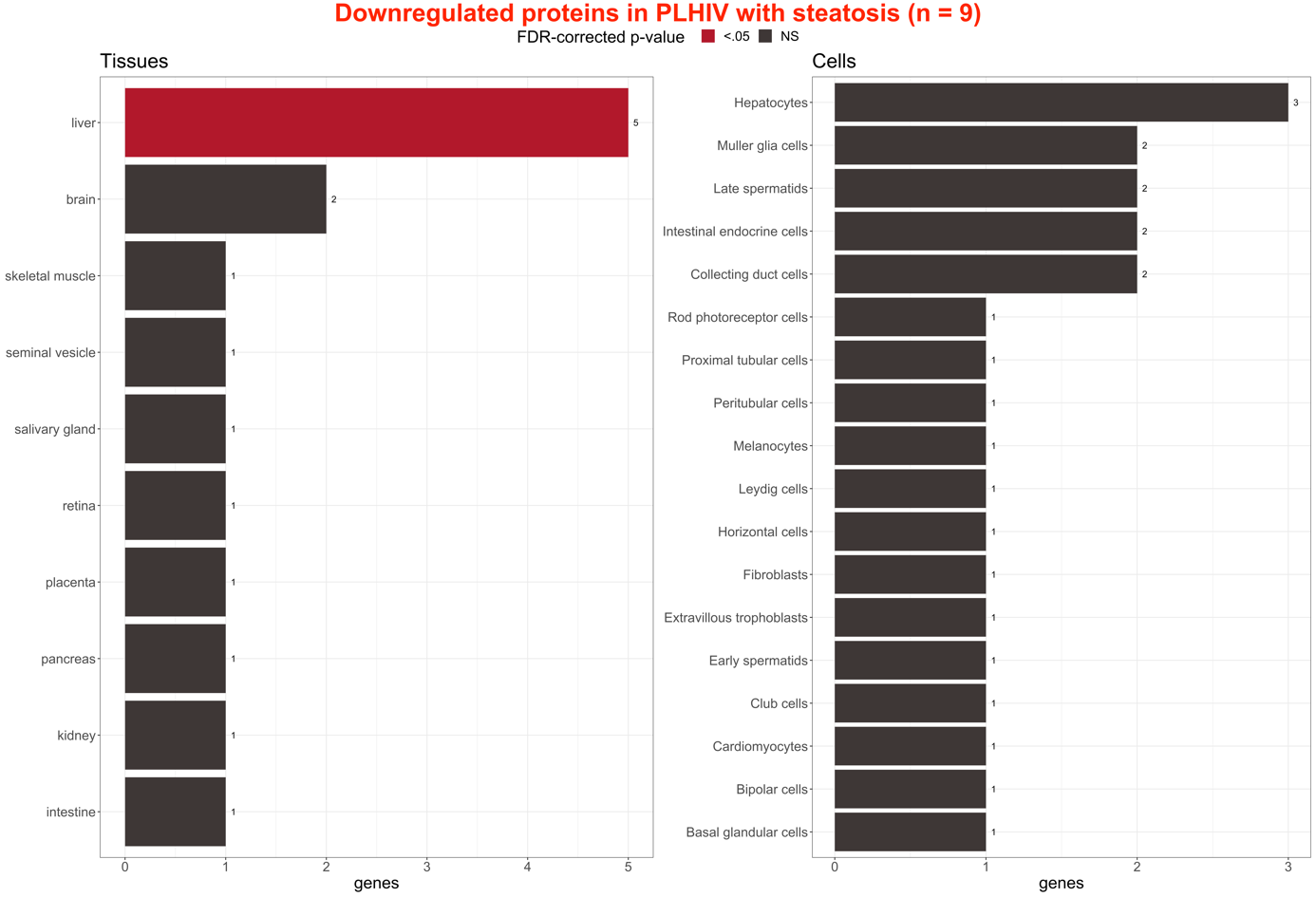


**Fig. S3** Tissues and cells enriched with differentially expressed proteins in PLHIV with steatosis compared to PLHIV without steatosis. Figure A-D show the enriched tissues (left) and cell types (right) for upregulated (figure A and B) and downregulated proteins (figure C and D).

**Fig. S4** Pathway enrichment analysis of the genes encoding for the upregulated DEPs associated with steatosis, using pathways of the Gene Ontology Biological Processes, KEGG pathways (n = 227) and Reactome gene sets (n = 765). The analysis was performed using the Metascape pathway enrichment analysis tool for multiple gene sets, applying the following settings: minimal overlap of three proteins, p-value cut-off of 0.05, and minimal enrichment of 1.5.

**Fig. S5** Protein-protein interaction network considering 67 differentially expressed proteins in PLHIV with steatosis (upper plots) and 17 differentially expressed proteins in PLHIV with fibrosis (lower plots). Separate networks are created for those with (right figures) and without (left figures) liver steatosis or fibrosis. The networks are based on Spearman correlation coefficients > 0.5. The weight of the connections between proteins refers to the correlation coefficients. The darker the red color, the stronger the correlation between proteins. The centrality, assessed using page rank centrality, is shown by yellow to dark blue colours; yellow for less central proteins, dark blue for more central proteins. IGSF9 is annotated with a red label in all plots.


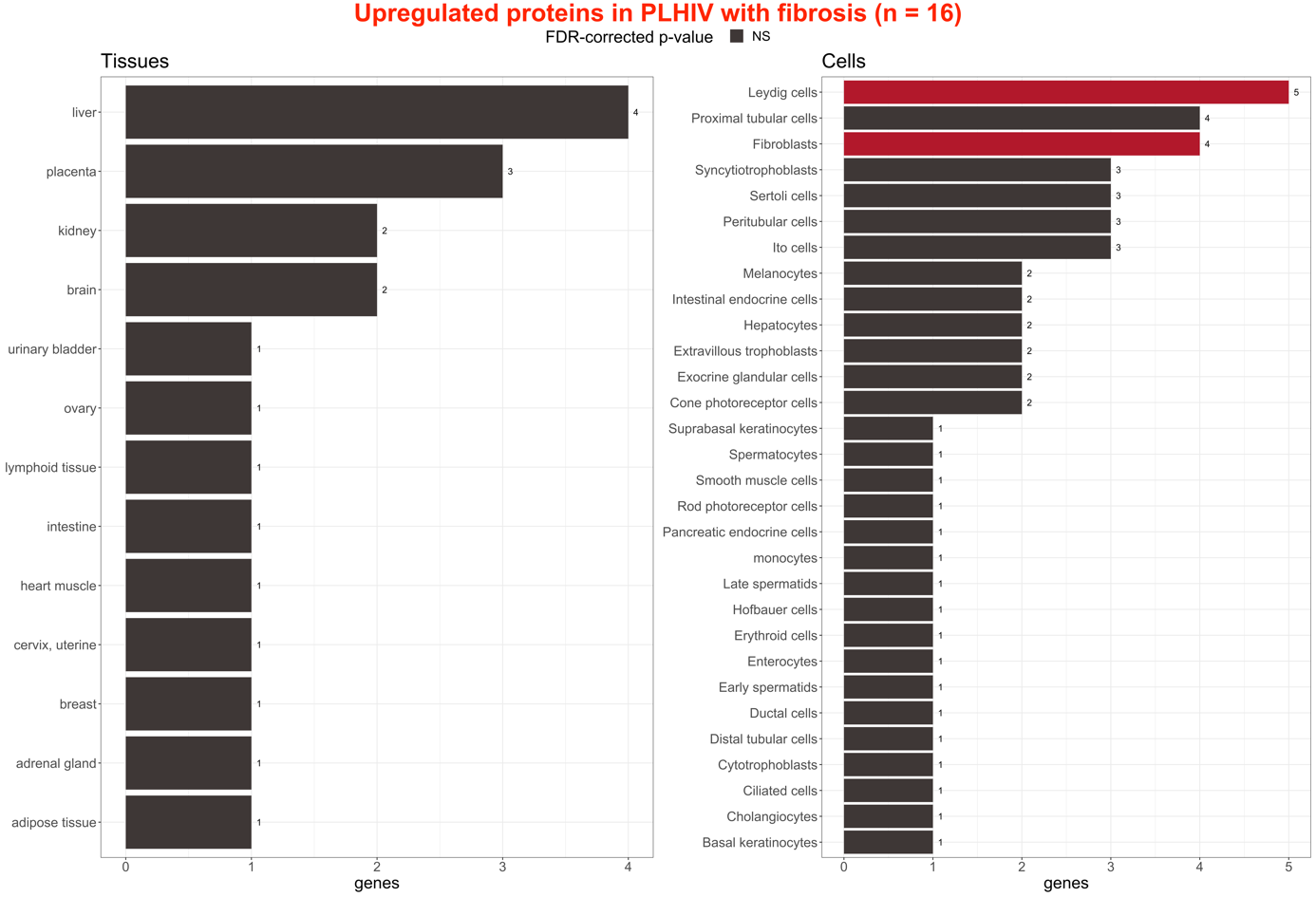


**Fig. S6** Tissues and cells enriched with differentially upregulated proteins in PLHIV with fibrosis compared to PLHIV without fibrosis. The left figure shows enriched tissues and the right figure shows the enriched cell types.
