# Supplementary material for: Plasma proteomic signatures of liver steatosis and fibrosis in people living with HIV: a cross-sectional study
Source: eBioMedicine. 2024 Oct 18;109:105407. doi: 10.1016/j.ebiom.2024.105407 (PMC11513669; doi:10.1016/j.ebiom.2024.105407)
Supplement: Graphical Abstract [file mmc3.pdf]

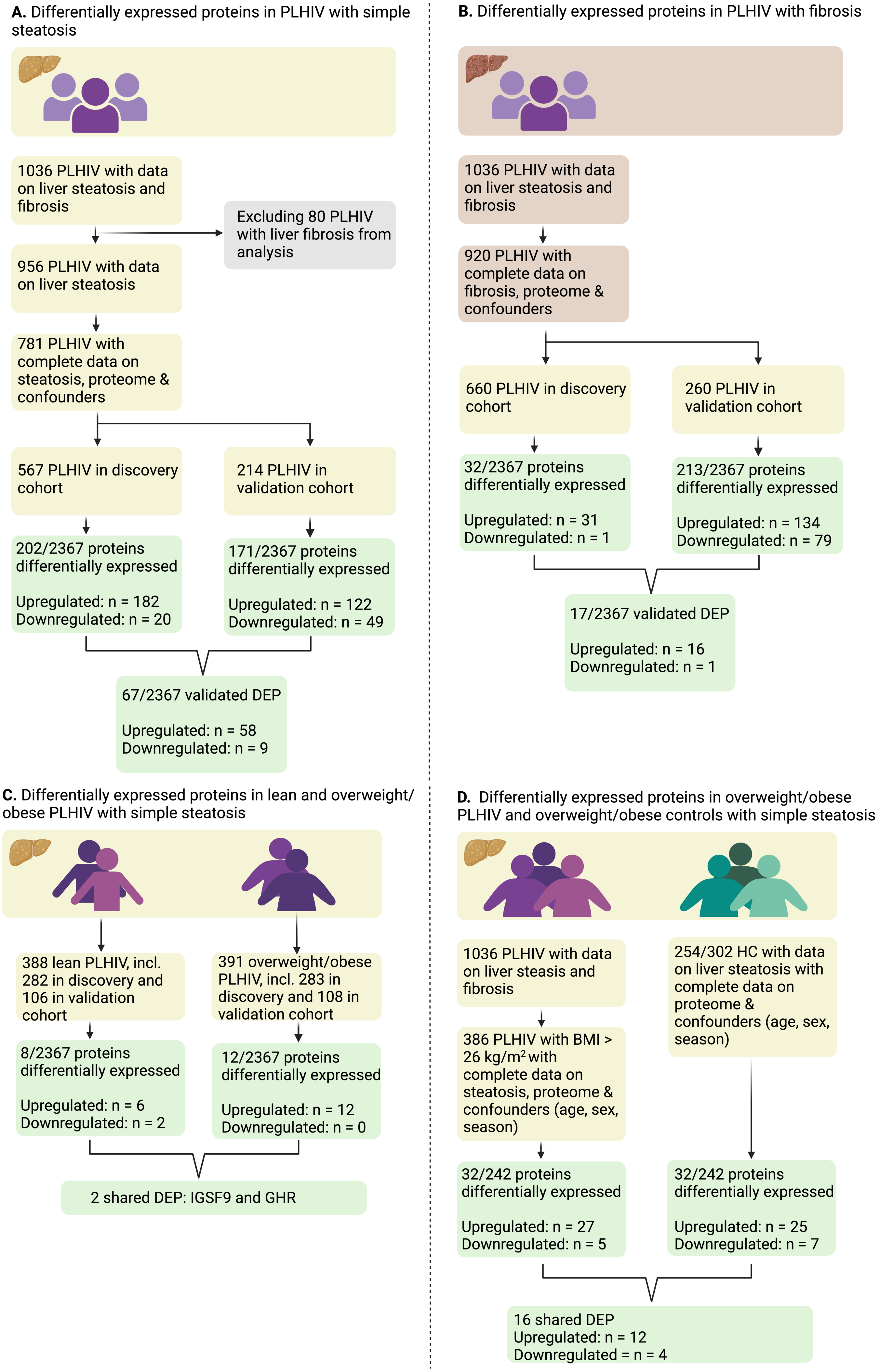

A. Differentially expressed proteins in PLHIV with simple steatosis
1036 PLHIV with data on liver steatosis and fibrosis
Excluding 80 PLHIV with liver fibrosis from analysis
956 PLHIV with data on liver steatosis
781 PLHIV with complete data on steatosis, proteome & confounders
567 PLHIV in discovery cohort
214 PLHIV in validation cohort
202/2367 proteins differentially expressed
Upregulated: n = 182
Downregulated: n = 20
171/2367 proteins differentially expressed
Upregulated: n = 122
Downregulated: n = 49
67/2367 validated DEP
Upregulated: n = 58
Downregulated: n = 9
B. Differentially expressed proteins in PLHIV with fibrosis
1036 PLHIV with data on liver steatosis and fibrosis
920 PLHIV with complete data on fibrosis, proteome & confounders
660 PLHIV in discovery cohort
260 PLHIV in validation cohort
32/2367 proteins differentially expressed
Upregulated: n = 31
Downregulated: n = 1
213/2367 proteins differentially expressed
Upregulated: n = 134
Downregulated: n = 79
17/2367 validated DEP
Upregulated: n = 16
Downregulated: n = 1
C. Differentially expressed proteins in lean and overweight/obese PLHIV with simple steatosis
388 lean PLHIV, incl. 282 in discovery and 106 in validation cohort
391 overweight/obese PLHIV, incl. 283 in discovery and 108 in validation cohort
8/2367 proteins differentially expressed
Upregulated: n = 6
Downregulated: n = 2
12/2367 proteins differentially expressed
Upregulated: n = 12
Downregulated: n = 0
2 shared DEP: IGSF9 and GHR
D. Differentially expressed proteins in overweight/obese PLHIV and overweight/obese controls with simple steatosis
1036 PLHIV with data on liver steasis and fibrosis
254/302 HC with data on liver steatosis with complete data on proteome & confounders (age, sex, season)
386 PLHIV with BMI > 26 kg/m^2 with complete data on steatosis, proteome & confounders (age, sex, season)
32/242 proteins differentially expressed
Upregulated: n = 27
Downregulated: n = 5
32/242 proteins differentially expressed
Upregulated: n = 25
Downregulated: n = 7
16 shared DEP
Upregulated: n = 12
Downregulated = n = 4
